# Differential molecular pathway expression according to chemotherapeutic response in ovarian clear cell carcinoma

**DOI:** 10.1186/s12905-023-02420-1

**Published:** 2023-06-03

**Authors:** Min Yin, Chunli Lu, Huimei Zhou, Qian Liu, Jiaxin Yang

**Affiliations:** 1 Department of Obstetrics and Gynecology, National Clinical Research Center for Obstetric and Gynecologic Diseases, Peking Union Medical College Hospital, Chinese Academy of Medical Sciences and Peking Union Medical College, Beijing, China; 2 Neurospine Center, Xuanwu Hospital, National Center for Neurological Disorders, China International Neuroscience Institute (CHINA-INI), Capital Medical University, Beijing, China

**Keywords:** Ovarian clear cell carcinoma, Gene expression profiling, Drug resistance, Platinum

## Abstract

**Objective:**

Ovarian clear cell carcinoma (OCCC) is a distinct entity from epithelial ovarian cancer. The prognosis of advanced and recurrent disease is very poor due to resistance to chemotherapeutic agents. Our aim was to explore the molecular alterations among OCCC patients with different chemotherapeutic responses and to obtain insights into potential biomarkers.

**Methods:**

Twenty-four OCCC patients were included in this study. The patients were divided into two groups based on the relapse time after the first-line platinum-based chemotherapy: the platinum-sensitive group (PS) and the platinum-resistant group (PR). Gene expression profiling was performed using NanoString nCounter PanCancer Pathways Panel.

**Results:**

Gene expression analysis comparing PR vs. PS identified 32 differentially expressed genes: 17 upregulated genes and 15 downregulated genes. Most of these genes are involved in the PI3K, MAPK and Cell Cycle-Apoptosis pathways. In particular, eight genes are involved in two or all three pathways.

**Conclusion:**

The dysregulated genes in the PI3K, MAPK, and Cell Cycle-Apoptosis pathways identified and postulated mechanisms could help to probe biomarkers of OCCC platinum sensitivity, providing a research basis for further exploration of targeted therapy.

**Graphic abstract:**

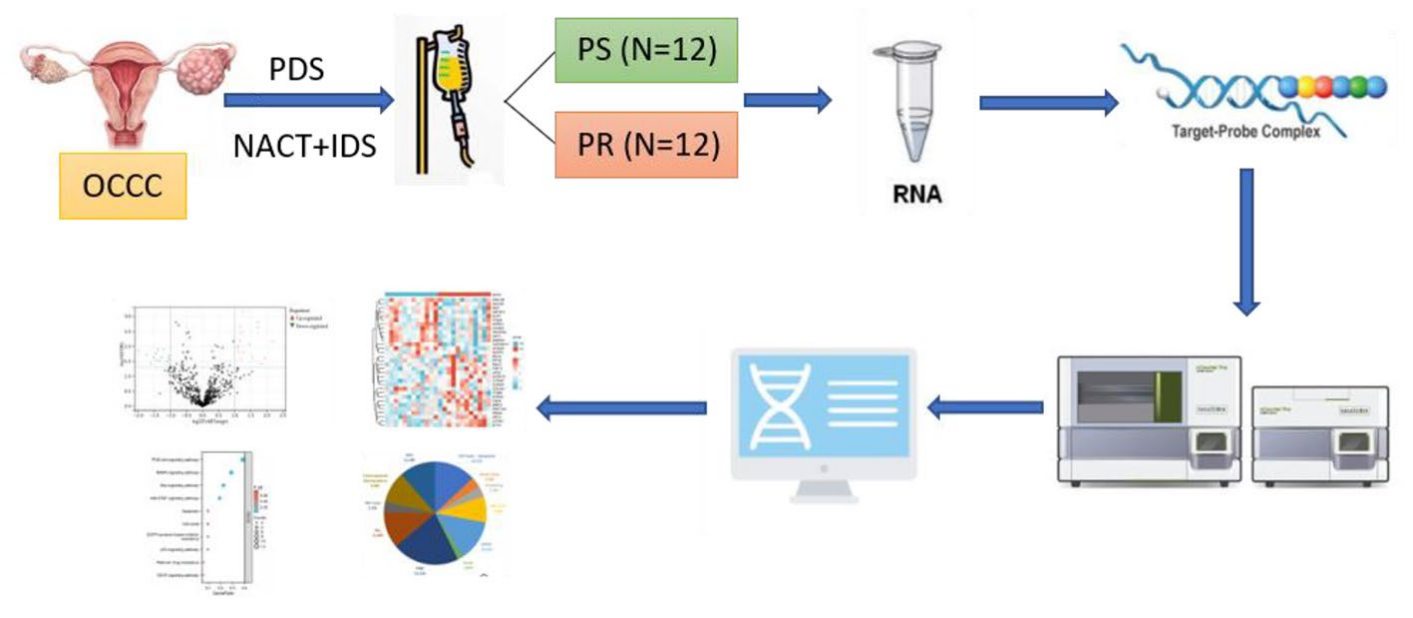

**Supplementary Information:**

The online version contains supplementary material available at 10.1186/s12905-023-02420-1.

## Introduction

Ovarian cancer is the third most common gynecologic malignancy worldwide but is the most lethal among these cancers [1]. Epithelial ovarian cancer (EOC) is the most common type of ovarian malignancy, accounting for over 95% of cases [2]. EOC can be divided into five main types based on histopathology and molecular features: high-grade serous (HGS) (70%), low-grade serous (< 5%), clear cell (10%), endometrioid (10%), and mucinous (3%) ovarian cancer. The different subtypes have unique patterns of clinical manifestation, therapeutic response and outcome [3]. Ovarian clear cell carcinoma (OCCC) represents approximately 4–12% of EOC in Western countries but is more prevalent in Asian countries, especially in Japan, where it occurs in almost 25% of EOC patients [4]. The median age at diagnosis of OCCC is younger than that of HGS, and OCCC has a high incidence of hypercalcemia and thromboembolic complications [5]. The pathogenesis of OCCC is not yet clear but the presence of endometriosis is an important risk factor [6].

Currently, there is no screening algorithm for asymptomatic women [7] but some strategies can help to discriminate malignant from benign lesions such as carbohydrate antigen 125 (CA125) [8], human epididymis protein 4 (HE4) [9], ultrasonography, risk of malignancy algorithm (ROMA), risk of ovarian cancer algorithm (ROCA), and risk of malignancy index (RMI) [10]. CA125 is increased in less than half of early-stage (stage I/II) or unilateral ovarian cancer cases and is more sensitive in patients with disseminated disease [7]. Therefore, CA125 is not very sensitive for OCCC because it is commonly diagnosed at an early stage (57-81%) [11]. Additionally, pretreatment CA125 levels were found not to be very useful for predicting clinical outcomes in OCCC, though the CA125 normalization time was shown to be associated with prognosis [12]. HE4 is reported to be sensitive for assessing hormonal treatment and robust for menstrual cycle variation; therefore, HE4 is potentially superior to CA125 as a marker for identifying women with endometriosis at risk of developing ovarian cancer [13]. Regarding the performance of RMI and ROMA in different histologic subtypes and stages of ovarian cancer, these triaging algorithms performed well for detection of advanced ovarian cancer and HGS histology, but did not perform well in patients with stage I disease, in which endometrioid and clear cell histologies predominate [14].

The current standard treatment for newly diagnosed OCCC is comprehensive staging surgery for early-stage disease and debulking surgery for advanced-stage disease. Achieving full cytoreduction and no residual disease has been shown to be a good prognostic indicator [15]. For unresectable disease, neoadjuvant chemotherapy (NACT) has been introduced to decrease tumor load and a unique complete surgery [16]. However, there is still controversy about the use of NACT in OCCC [17, 18]. Postoperatively, chemotherapy with paclitaxel and carboplatin (TC) is recommended for all patients with stage IC2 and above [19–21].

In comparison to other subtypes of EOC, OCCC is relatively resistant to conventional platinum-based chemotherapy. Indeed, the response rate of OCCC to platinum-based chemotherapy is reportedly 11-50%, less than that of HGS (73-81%) [22]. Moreover, the response rate in the relapse setting is as low as 6-8% [23]. Therefore, OCCC outcomes are much worse in stage III/IV and recurrence. Several mechanisms of chemoresistance of OCCC have been proposed, including drug efflux, drug inactivation, and an increase in DNA repair activity [24]. Other mechanisms involving annexin A4, metabolic alterations caused by hepatocyte nuclear factor-1 beta (HNF1B), mitochondrial function, the insulin like growth factor 1 receptor (IGF1R)/AKT pathway, and the caveolin-1/angiotensin-converting enzyme 2 (ACE2) axis have also been proposed [25–30]. In addition, it has been reported that some somatic mutations are highly frequent in OCCC, such as in *AT-rich interaction domain 1 A (ARID1A)*, *phosphatidylinositol‐4,5‐bisphosphate 3‐kinase catalytic subunit alpha (PIK3CA)*, and *phosphatase, tensin homolog (PTEN)* [5, 31]. Genetic modifications that alter gene expression have an impact on downstream molecular pathways and result in aberrant cell function and progression of OCCC [3, 32]. Overall, comparing differential gene expression profiles in the development of chemoresistance will facilitate identification of possible biomarkers for predicting chemosensitivity and potential therapeutic targets.

In this study, we used NanoString nCounter PanCancer Pathways Panel to explore expression of genes and molecular pathways responsible for conferring the disparity between platinum-sensitivity and platinum-resistance groups and to obtain insights into potential biomarkers.

## Materials and methods

### Patient selection and ethics

Patients who underwent surgery and were diagnosed with OCCC at International Federation of Gynecology and Obstetrics (FIGO) stage II-IV from January 2019 to December 2021 were examined. Exclusion criteria were as follows: (1) mixed subtypes diagnosed by histopathology; (2) not receiving standardized platinum-based adjuvant chemotherapy postoperatively; and (3) complicated with chronic system diseases or other malignant tumors. Medical data, including age, preoperative CA125, NACT, surgical approach, FIGO stage, residual disease, chemotherapy regimen, and chemotherapy cycles, were collected. Patients were divided into two groups based on whether the relapse time after the first-line platinum-based chemotherapy exceed 6 months: the platinum-sensitive group (PS) and the platinum-resistant group (PR). The PS group served as the control group. The FIGO stage, NACT, and residual disease were matched in frequency between the two groups. Finally, 24 patients were selected, with 12 patients in each group. The study was conducted in accordance with the Declaration of Helsinki and approved by the Institutional Review Board of Peking Union Medical College Hospital (JS-1747). Informed consent was obtained from all subjects involved in the study.

### Sample collection and processing

Formalin-fixed paraffin-embedded (FFPE) blocks were obtained from the archives of the department of pathology. Two experienced pathologists reviewed hematoxylin and eosin (H&E)-stained sections and identified the presence of tumors in a slide from the block. Five 10 μm curls were then cut from each tumor subblock.

### Total RNA extraction and quality assurance

Total RNA was isolated from the curls using RNeasy Mini Kit (Qiagen, Germany) according to the manufacturer’s protocol. The RNA quality was assessed using an Agilent 2100 Bioanalyzer (Agilent, USA) and NanoDrop (Thermo Scientific, USA). The A260/280 and A260/230 ratios from the spectrophotometer results were used to assess the purity of the isolated RNA. The A260/280 ratio should be more than 2.1, and the A260/230 ratio should be more than 1.8. The RNA concentration of the samples should be more than 300 ng/µL.

### Nanostring ncounter pancancer pathways panel detection

Gene expression profiling was performed using the NanoString nCounter PanCancer Pathways Panel kit (NanoString Technologies, USA) following the manufacturer’s recommendations. First, 3 µL reporter CodeSet and 5 µL hybridization buffer were mixed to create a hybridization master mix. Then, 5 µL of sample was added to each tube containing the hybridization master mix and 2 µL of Capture ProbeSet was added to each tube. After brief centrifugation, the tubes were immediately placed in a preheated 65 °C thermal cycler for 16–24 h. The tubes were removed from the thermal cycler and immediately loaded onto nCounter Prep Station. After two rounds of magnetic bead purification, the sample cartridges were placed on a digital analyzer and scanned by a fluorescence microscope. For each target molecule, the barcodes were counted, and the data were exported as a CSV file.

### Gene expression profiling analysis

Data analysis was performed using nSolver 4.0 software; the detailed workflow for data analysis can be found in nSolver Analysis Software User Manual (https://www.nanostring.com/products/analysis-software/nsolver). Using R-Project software, volcano plots were drawn based on the fold change (FC) and the P value of the test between the two groups of samples. Hierarchical cluster analysis was used to preliminarily classify the results from two dimensions: sample and gene differential expression patterns. Gene Ontology (GO) and Kyoto Encyclopedia of Genes and Genomes (KEGG) pathway enrichment analyses of differentially expressed genes (DEGs) were performed to explore biological functions between the two groups.

### Statistical analysis

Gene expression comparisons were performed between the PR and PS groups. As log_2_-transformed count data were normally distributed, the significance of gene expression was determined using a t-test with log_2_-transformed count data and statistical significance set at P value < 0.05. The screening criteria for DEGs were FC > 2 and P value < 0.05.

## Results

### Clinicopathological characteristics of the patients

We evaluated a total of 24 archived FFPE OCCC tissue samples. After total RNA extraction and RNA quality checks, all samples were adequate for NanoString nCounter analysis. Additional File Table S1 lists the detailed information of the RNA quality check. The detailed clinicopathological features of the two groups are shown in Table 1. FIGO stage, NACT, and residual disease were matched in frequency across the two groups. All patients received TC chemotherapy except one patient experienced an allergic reaction to paclitaxel and was subsequently treated with cisplatin and cyclophosphamide.

**Table 1:** Clinicopathological features of the patients

Patient ID	Age	Preoperative CA125 (U/mL)	NACT	FIGO stage	Residual disease	Chemotherapy regimen	Chemotherapy cycles
OCCC-PR-01	51	1013	-	III	R0	TC	6
OCCC-PR-02	52	186.2	-	III	R1	TC	6
OCCC-PR-03	35	82.2	-	II	R0	TC	6
OCCC-PR-04	47	11.5	-	II	R0	TC	6
OCCC-PR-05	47	147.1	-	III	R0	TC	6
OCCC-PR-06	41	68.3	TC×3	IV	R2	TC	6
OCCC-PR-07	50	123.4	-	II	R0	TC	6
OCCC-PR-08	52	598.2	-	III	R0	TC	6
OCCC-PR-09	32	302	-	III	R0	TC	6
OCCC-PR-10	43	555.9	TC×1	III	R2	TC	6
OCCC-PR-11	58	390	-	III	R1	TC	6
OCCC-PR-12	31	260	-	II	R0	DDP + CTX*	1 + 6
OCCC-PS-01	65	204.3	-	II	R0	TC	6
OCCC-PS-02	52	115.8	-	II	R0	TC	6
OCCC-PS-03	57	11.8	-	III	R0	TC	6
OCCC-PS-04	51	54.7	-	III	R1	TC	6
OCCC-PS-05	42	30.5	-	II	R0	TC	6
OCCC-PS-06	58	229	-	III	R1	TC	6
OCCC-PS-07	51	29.3	-	III	R0	TC	6
OCCC-PS-08	51	43.3	-	III	R1	TC	6
OCCC-PS-09	51	1952	TC×3	IV	R2	TC	6
OCCC-PS-10	43	69.3	-	III	R0	TC	6
OCCC-PS-11	27	45	-	II	R0	TC	6
OCCC-PS-12	51	59	TC×2	III	R2	TC	6

Abbreviations: NACT, neoadjuvant chemotherapy; R, residual disease; R0, no macroscopic residual disease; R1, macroscopic residual disease with a maximal diameter < 1 cm; R2, macroscopic residual disease with a maximal diameter > 1 cm; TC, paclitaxel and carboplatin; DDP, cisplatin; CTX, cyclophosphamide

* This patient experienced an allergic reaction to paclitaxel and was subsequently treated with cisplatin and cyclophosphamide for 6 cycles

### DEGs between PR and PS groups

**Fig. 1:**
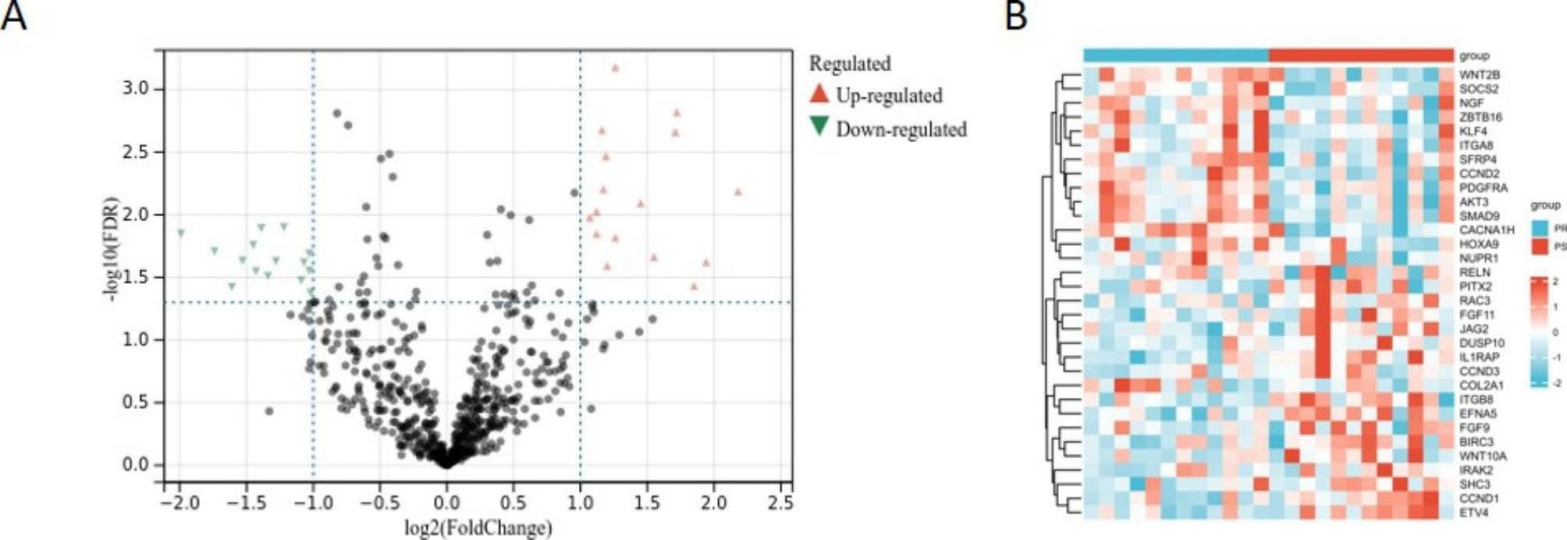
(A) Volcano plot showing differentially expressed genes (DEGs) of the PR vs. PS group. Screening criteria for DEGs were fold change > 2 and P value < 0.05. (B) Hierarchical clustering based on mRNA expression levels of these 32 DEGs

NanoString nCounter PanCancer Pathways Panel is a multiplex gene expression panel that includes 770 genes from 13 canonical pathways and selected reference genes. The 13 cancer-related pathways are Cell Cycle-Apoptosis, Chromatin Modification, DNA Damage-Repair, Driver Gene, Hedgehog, JAK-STAT, MAPK, Notch, PI3K, Ras, TGF-beta, Transcriptional Misregulation, and Wnt. Thirty-two DEGs were identified in the PR group compared with the PS group, with a FC > 2 and P < 0.05 (Additional File Table S2). As shown in Fig. 1A, seventeen genes were significantly upregulated and fifteen genes downregulated. Hierarchical clustering based on the mRNA expression levels of these 32 DEGs is visualized in Fig. 1B.

### GO and KEGG enrichment analyses of DEGs

**Fig. 2:**
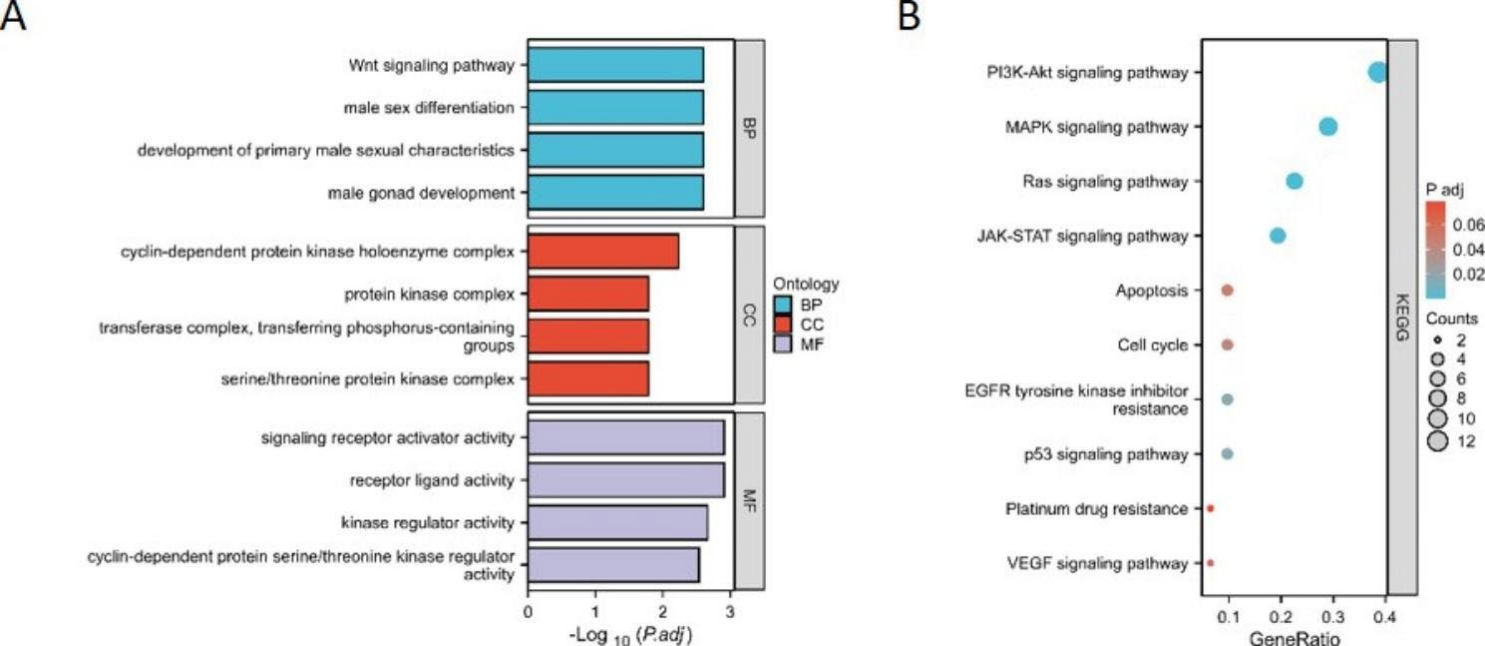
Functional enrichment analysis of differentially expressed genes (DEGs) using GO and KEGG. (A) GO enrichment analysis of DEGs. (B) KEGG enrichment analysis of DEGs. GO, Gene Ontology; KEGG, Kyoto Encyclopedia of Genes and Genomes; BP, biological process; CC, cell component; MF, molecular function

We performed GO and KEGG analyses to determine the molecular functions and pathways in which these DEGs are involved. GO analysis of 32 DEGs showed that they are closely related to the following molecular functions: signaling receptor activator activity, receptor ligand activity, kinase regulator activity, and cyclin-dependent protein serine/threonine kinase regulator activity (Fig. 2A). For KEGG pathway enrichment, DEGs were mainly enriched in the PI3K/AKT signaling pathway, MAPK signaling pathway, Ras signaling pathway, JAK-STAT signaling pathway, apoptosis and cell cycle (Fig. 2B).

### Gene set analysis

**Fig. 3:**
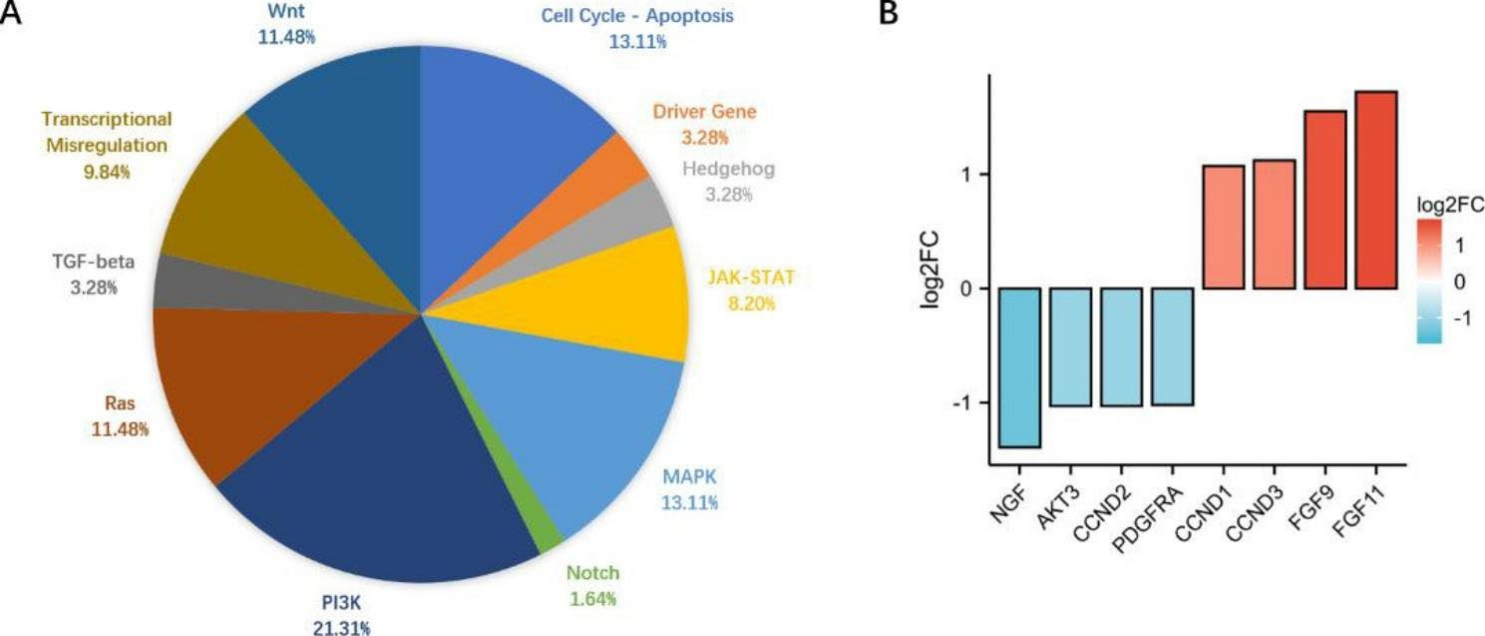
(A) Distribution of the 32 DEGs at the gene set level. (B) Genes differentially expressed between the PR and PS involved in two or all the PI3K, MAPK, and Cell Cycle-Apoptosis pathways. *NGF*, nerve growth factor; *AKT3*, v-akt murine thymoma viral oncogene homolog 3; *CCND2*, cyclin D2; *PDGFRA*, platelet-derived growth factor receptor, alpha polypeptide; *CCND1*, cyclin D1; *CCND3*, cyclin D3; *FGF9*, fibroblast growth factor 9; *FGF11*, fibroblast growth factor 11

The analyzed genes were classified into 13 canonical cancer-related pathways. according to the gene set defined by the NanoString nCounter PanCancer Pathways Panel. The list of genes expressed in each pathway is shown in Additional File Table S2. Most of the DEGs are involved in the PI3K pathway (21.31%), MAPK pathway (13.11%), and Cell Cycle-Apoptosis pathway (13.11%) (Fig. 3A). Some of the DEGs engage in crosstalk between various signaling pathways, playing an important role in modulation of signal transduction and drug resistance. In particular, eight genes are involved in two or all three pathways (Fig. 3B; Table 2). *Fibroblast growth factor (FGF)11* was highly upregulated, with a log_2_FC of 1.72, followed by *FGF9*, at 1.55. *NGF (nerve growth factor)* was highly downregulated with a log_2_FC of -1.39.

**Table 2:** Eight genes involved in the PI3K, MAPK, and Cell Cycle-Apoptosis pathways

Gene	Official Full Name	Log_2_FC	P value	Gene set
*NGF*	nerve growth factor	-1.39	0.0128	Cell Cycle-Apoptosis, MAPK, PI3K, Ras
*AKT3*	v-akt murine thymoma viral oncogene homolog 3	-1.03	0.0205	Cell Cycle-Apoptosis, JAK-STAT, MAPK, PI3K, Ras
*CCND2*	cyclin D2	-1.03	0.028	Cell Cycle-Apoptosis, JAK-STAT, PI3K, Transcriptional Misregulation, Wnt
*PDGFRA*	platelet-derived growth factor receptor, alpha polypeptide	-1.02	0.042	Driver Gene, MAPK, PI3K, Ras
*CCND1*	cyclin D1	1.07	0.0105	Cell Cycle-Apoptosis, JAK-STAT, PI3K, Wnt
*CCND3*	cyclin D3	1.12	0.0142	Cell Cycle-Apoptosis, JAK-STAT, PI3K, Wnt
*FGF9*	fibroblast growth factor 9	1.55	0.0217	MAPK, PI3K, Ras
*FGF11*	fibroblast growth factor 11	1.72	0.00152	MAPK, PI3K, Ras

## Discussion

OCCC shows a low response rate to platinum-based chemotherapy. Consequently, the clinical prognosis of advanced and recurrent OCCC is remarkably low, warranting development of novel biomarkers and targeted therapies [25]. Chemoresistance in ovarian cancer involves multifaceted mechanisms that are associated with a number of genes and signaling pathways [33]. This exploratory study focused on comparing gene expression profiles between PR and PS OCCC patients using FFPE tissues. Most of the DEGs obtained are involved in the PI3K, MAPK and Cell Cycle-Apoptosis pathways. Eight genes are involved in two or all three pathways and are potential biomarkers of the chemotherapeutic response and targets for overcoming OCCC chemoresistance.

### PI3K pathway

Abnormalities in the PI3K/AKT/mTOR signaling pathway are very prevalent in malignant tumors and mutations in *PIK3CA* have been frequently detected in many cancers including OCCC, reportedly at 40% [34]. Preclinical studies have shown that some inhibitors of this pathway can inhibit progression of OCCC [35–37]; several clinical studies have been completed but have shown a low objective response rate for these inhibitors when used as single agents [38, 39]. Bevacizumab, a humanized monoclonal antibody that targets vascular endothelial growth factor A (VEGFA), was licensed as a maintenance therapy following first-line chemotherapy in EOC based on the results of the ICON7 clinical trial [40, 41]. Studies have shown that adding bevacizumab to first-line chemotherapy for OCCC improved progression-free survival at advanced stage [42] and that adding bevacizumab to chemotherapy for recurrent OCCC is also effective [43, 44].

**Fig. 4:**
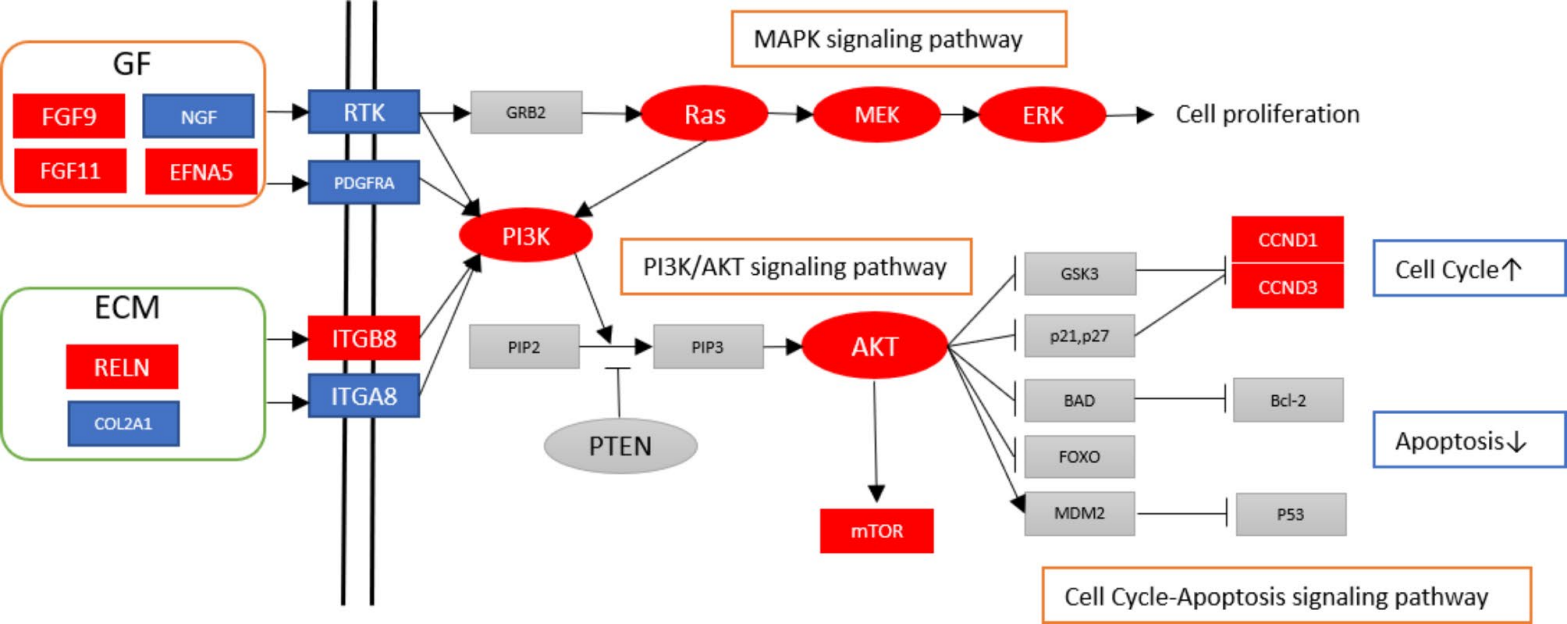
Postulated mechanisms involved in platinum-resistant OCCC. Red box/circle: significantly upregulated gene; blue box, significantly downregulated gene; gray box/circle: not significantly expressed gene or not included in this panel. GF, growth factor; FGF, fibroblast growth factor; NGF, nerve growth factor; EFNA5, ephrin-A5; ECM, extracellular matrix; RELN, reelin; RTK, receptor tyrosine kinase; PDGFRA, platelet-derived growth factor receptor, alpha polypeptide; ITGB8, integrin, beta 8; ITGA8, integrin, alpha 8; GRB2, growth factor receptor-bound protein-2; GSK3, glycogen synthase kinase-3; CCND1, cyclin D1; CCDN3, cyclin D3

The tumor microenvironment (TME) comprises extracellular components (cytokines, growth factors, extracellular matrix, etc.) as well as different cell types (fibroblasts, immune cells, etc.). Interactions between tumor cells and their surrounding stroma result in environment-mediated treatment resistance [45]. Recently, a study described a comprehensive analysis of the TME of OCCC and found that advanced-stage disease had significantly more fibroblasts and a more complex collagen matrix than early OCCC [46]. In the present study, we found that several growth factors including FGF9, FGF11, and ephrin-A5 (EFNA5) were significantly upregulated and associated with the PI3K pathway (Fig. 4). Fibroblast growth factors belong to a large family of growth factors, that mediate a wide range of biological and pathological processes via paracrine or endocrine signaling. FGF/FGFR signaling plays crucial roles in cancer development [47]. However, the role of FGFs in OCCC remains to be explored.

In addition, activation of PI3K or inactivation of PTEN induces an immune-suppressive state in the TME [32], showing that OCCC has a unique immune microenvironment; thus, immunotherapy may be an attractive strategy. Tumor-infiltrating lymphocytes (TILs) were shown to be linked to greater chemosensitivity and better prognosis in ovarian cancer [48]. However, concerning different histological subtypes, the highest presence of TILs is in HGS, whereas mucinous and OCCC appear to include a smaller percentage of TILs [49, 50]. In particular, recent clinical trials of immune checkpoint inhibitors reported a higher response rate in patients with advanced or recurrent OCCC, supporting further investigation of immune checkpoint inhibitors in OCCC [51–53].

### MAPK pathway

The MAPK pathway is a highly conserved signal transduction cascade that is situated downstream of many growth factors receptors. Therefore, the MAPK pathway is activated by various stimuli, including peptide growth factors, cytokines, and hormones, among others, regulating cell proliferation, migration, apoptosis, and differentiation [54]. MAPK-related changes are most frequently discovered in the oncogene *KRAS*, which is overexpressed in 18% of OCCCs and mutated in 5–14% of cases [54]. Due to the high frequency of genetic changes via the MAPK pathway in OCCC, preclinical and clinical efforts have been directed toward investigating the efficacy of MAPK pathway inhibition as treatment. One study showed that a low-dose triple drug combination targeting the PI3K/AKT/mTOR pathway and the MAPK pathway significantly reduced tumor growth in two patient-derived xenograft (PDX) models [55]. Thus, inhibiting the MAPK pathway may be feasible for treating OCCC, and more studies and clinical trials are needed.

### Cell cycle-apoptosis pathway

Cyclins D1, D2, and D3 function together as allosteric regulators of cyclin-dependent kinase 4 and 6 (CDK4/CDK6) to control progression of the cell cycle from G1 to S phase [56]. Previous work revealed that PI3K/AKT regulated CCND1 nuclear accumulation through modulation of GSK3β, promoting G1/S transition [57]. In the present study, we found that CCND1 and CCND3 were overexpressed in platinum-resistant OCCC, leading to rapid cell proliferation with constrained mitogenic signaling, which is consistent with a previous report [58]. Although cyclin D1 lacks enzymatic activity, its catalytic partners CDK4/CDK6 can be highly specifically targeted [59]. The CDK4/CDK6 inhibitors palbociclib and abemaciclib have been approved for treatment of advanced or metastatic breast cancer [60]. These inhibitors are also being studied extensively in a variety of clinical trials, including in ovarian cancer.

Apoptosis occurs through one of two mechanisms: an extrinsic pathway that is receptor dependent and initiated outside the cell, or an intrinsic pathway that is mitochondrion dependent [61]. Most anticancer pharmaceuticals induce apoptosis and associated cell death. In cancer, dysregulated apoptotic signaling, particularly activation of anti-apoptotic systems, allows cancer cells to escape from apoptosis, resulting in tumor survival, therapeutic resistance, and cancer recurrence [62]. In ovarian cancer, PI3K/AKT pathway inhibits induction of apoptosis-related proteins, therefore increasing cisplatin resistance (Fig. 4). Alkylating drugs, including carboplatin and cisplatin, bind to DNA to create intra- and interstrand crosslinks, resulting in DNA damage that leads to mitochondrion-mediated apoptosis [63]. Identifying key modulators of apoptosis may serve as a basis for development of new treatment modalities. Second mitochondria-derived activator of caspase (SMAC) has been described as sensitizing cells to apoptosis. A preclinical study showed that the small molecule SMAC mimic LBW242 strongly synergized with tumor necrosis factor-related apoptosis inducing ligand (TRAIL) or anticancer drugs to induce apoptosis of ovarian cancer cells by activating caspase-8 [64].

## Conclusion

In conclusion, dysregulated genes in the PI3K, MAPK, and Cell Cycle-Apoptosis pathways and postulated mechanisms described herein may help to identify biomarkers of OCCC platinum sensitivity, providing a research basis for further exploration of targeted therapy.

## Electronic supplementary material

Below is the link to the electronic supplementary material.

Additional File Table 1: Study sample details of RNA quality check

Additional File Table 2: List of 32 differentially expressed genes in PR vs. PS

## Data Availability

The datasets presented in this study can be found in the online repository. https://www.ncbi.nlm.nih.gov/geo/ under accession number GSE212670.
