# Supplementary material for: Differential molecular pathway expression according to chemotherapeutic response in ovarian clear cell carcinoma
Source: BMC Womens Health. 2023 Jun 3;23:298. doi: 10.1186/s12905-023-02420-1 (PMC10239578; doi:10.1186/s12905-023-02420-1)
Supplement: Supplementary file 1 — Additional File Table 1: Study sample details of RNA quality check [file 12905_2023_2420_MOESM1_ESM.docx]

Table S1. Study sample details of RNA quality check.

| Sample ID | Concentration (ng/µL) | A260/280 | A260/230 |
| --- | --- | --- | --- |
| OCCC-PS-01 | 321.1 | 2.11 | 1.82 |
| OCCC-PS-02 | 342.6 | 2.10 | 1.92 |
| OCCC-PS-03 | 423.5 | 2.14 | 1.91 |
| OCCC-PS-04 | 367.5 | 2.13 | 1.86 |
| OCCC-PS-05 | 353.6 | 2.12 | 1.94 |
| OCCC-PS-06 | 367.6 | 2.15 | 1.83 |
| OCCC-PS-07 | 425.4 | 2.16 | 1.97 |
| OCCC-PS-08 | 463.5 | 2.19 | 1.89 |
| OCCC-PS-09 | 388.3 | 2.12 | 1.98 |
| OCCC-PS-10 | 306.3 | 2.15 | 1.95 |
| OCCC-PS-11 | 310.5 | 2.12 | 1.93 |
| OCCC-PS-12 | 420.4 | 2.17 | 1.82 |
| OCCC-PR-01 | 324.5 | 2.14 | 1.97 |
| OCCC-PR-02 | 346.5 | 2.14 | 1.95 |
| OCCC-PR-03 | 754.3 | 2.18 | 1.90 |
| OCCC-PR-04 | 364.7 | 2.16 | 1.84 |
| OCCC-PR-05 | 424.3 | 2.18 | 1.96 |
| OCCC-PR-06 | 364.1 | 2.13 | 1.83 |
| OCCC-PR-07 | 357.4 | 2.13 | 1.96 |
| OCCC-PR-08 | 352.6 | 2.18 | 1.93 |
| OCCC-PR-09 | 421.3 | 2.14 | 1.87 |
| OCCC-PR-10 | 452.3 | 2.14 | 1.84 |
| OCCC-PR-11 | 547.4 | 2.15 | 1.93 |
| OCCC-PR-12 | 462.3 | 2.19 | 1.96 |
