# Supplementary material for: Differential molecular pathway expression according to chemotherapeutic response in ovarian clear cell carcinoma
Source: BMC Womens Health. 2023 Jun 3;23:298. doi: 10.1186/s12905-023-02420-1 (PMC10239578; doi:10.1186/s12905-023-02420-1)
Supplement: Supplementary file 2 — Additional File Table 2: List of 32 differentially expressed genes in PR vs. PS [file 12905_2023_2420_MOESM2_ESM.docx]

Table S2. List of 32 differentially expressed genes in PR vs. PS

| **Gene** | **Official Full Name** | **Log_2_FC** | **P value** | **Gene sets** |
| --- | --- | --- | --- | --- |
| *ZBTB16* | zinc finger and BTB domain containing 16 | -1.99 | 0.0142 | Transcriptional Misregulation |
| *SFRP4* | secreted frizzled-related protein 4 | -1.74 | 0.0196 | Wnt |
| *ITGA8* | integrin, alpha 8 | -1.61 | 0.0378 | PI3K |
| *HOXA9* | homeobox A9 | -1.53 | 0.0233 | Transcriptional Misregulation |
| *COL2A1* | collagen, type II, alpha 1 | -1.45 | 0.0175 | PI3K |
| *KLF4* | Kruppel-like factor 4 | -1.43 | 0.0283 | Driver Gene |
| *NGF* | nerve growth factor | -1.39 | 0.0128 | Cell Cycle-Apoptosis, MAPK, PI3K, Ras |
| *NUPR1* | nuclear protein, transcriptional regulator, 1 | -1.34 | 0.0309 | Transcriptional Misregulation |
| *SMAD9* | SMAD family member 9 | -1.28 | 0.0235 | TGF-beta |
| *CACNA1H* | calcium channel, voltage-dependent, T type, alpha 1H subunit | -1.22 | 0.0126 | MAPK |
| *WNT2B* | wingless-type MMTV integration site family, member 2B | -1.09 | 0.0334 | Hedgehog, Wnt |
| *SOCS2* | suppressor of cytokine signaling 2 | -1.07 | 0.0241 | JAK-STAT |
| *AKT3* | v-akt murine thymoma viral oncogene homolog 3 | -1.03 | 0.0205 | Cell Cycle-Apoptosis, JAK-STAT, MAPK, PI3K, Ras |
| *CCND2* | cyclin D2 | -1.03 | 0.028 | Cell Cycle-Apoptosis, JAK-STAT, PI3K, Transcriptional Misregulation, Wnt |
| *PDGFRA* | platelet-derived growth factor receptor, alpha polypeptide | -1.02 | 0.042 | Driver Gene, MAPK, PI3K, Ras |
| *CCND1* | cyclin D1 | 1.07 | 0.0105 | Cell Cycle-Apoptosis, JAK-STAT, PI3K, Wnt |
| *CCND3* | cyclin D3 | 1.12 | 0.0142 | Cell Cycle-Apoptosis, JAK-STAT, PI3K, Wnt |
| *JAG2* | jagged 2 | 1.12 | 0.00943 | Notch |
| *ITGB8* | integrin, beta 8 | 1.16 | 0.0021 | PI3K |
| *RAC3* | ras-related C3 botulinum toxin substrate 3 (rho family, small GTP binding protein Rac3) | 1.17 | 0.00623 | MAPK, Ras, Wnt |
| *EFNA5* | ephrin-A5 | 1.19 | 0.00341 | PI3K, Ras |
| *IRAK2* | interleukin-1 receptor-associated kinase 2 | 1.2 | 0.0255 | Cell Cycle-Apoptosis |
| *IL1RAP* | interleukin 1 receptor accessory protein | 1.26 | 0.000662 | Cell Cycle-Apoptosis |
| *BIRC3* | baculoviral IAP repeat containing 3 | 1.26 | 0.0152 | Cell Cycle-Apoptosis, Transcriptional Misregulation |
| *DUSP10* | dual specificity phosphatase 10 | 1.45 | 0.00807 | MAPK |
| *FGF9* | fibroblast growth factor 9 | 1.55 | 0.0217 | MAPK, PI3K, Ras |
| *ETV4* | ets variant 4 | 1.71 | 0.00218 | Transcriptional Misregulation |
| *FGF11* | fibroblast growth factor 11 | 1.72 | 0.00152 | MAPK, PI3K, Ras |
| *RELN* | reelin | 1.85 | 0.0369 | PI3K |
| *SHC3* | SHC (Src homology 2 domain containing) transforming protein 3 | 1.94 | 0.0238 | Ras |
| *PITX2* | paired-like homeodomain 2 | 2.18 | 0.00649 | TGF-beta |
| *WNT10A* | wingless-type MMTV integration site family, member 10A | 2.46 | 0.00276 | Hedgehog, Wnt |
